# Phytochemical Analysis and Central Effects of *Annona Muricata* Linnaeus: Possible Involvement of the Gabaergic and Monoaminergic Systems

**Published:** 2018

**Authors:** Daniele Oliveira Souza, Valterlúcio dos Santos Sales, Cristina Kelly de Souza Rodrigues, Larissa Rolim de Oliveira, Izabel Cristina Santiago Lemos, Gyllyandeson de Araújo Delmondes, Álefe Brito Monteiro, Emmily Petícia do Nascimento, Francisco Rodolpho Sobreira Dantas Nóbrega de Figuêiredo, José Galberto Martins da Costa, Giovany Michely Pinto da Cruz, Glauce Socorro de Barros Viana, Roseli Barbosa, Irwin Rose Alencar de Menezes, Cícero Francisco Bezerra Felipe, Marta Regina Kerntopf

**Affiliations:** a *Laboratory of Molecular Chemistry and Pharmacology, Regional University of Cariri Antonio Luis, Crato (CE), Brazil. *; b *Laboratory of Research in Natural Products, Regional University of Cariri Antonio Luis, Crato (CE), Brazil.*; c *Laboratory of Pharmacology and Biophysiology, Faculty of Medicine Estácio of Juazeiro do Norte, 515 Tenente Raimundo Rocha, Juazeiro do Norte (CE), Brazil.*; d *Laboratory of Pharmacology and Experimental Biochemistry, Federal University of Paraíba, Cidade Universitária Pessoa (PB), Brazil.*

**Keywords:** Annona muricata, Sedative, Anxiolytic, Anticonvulsant, GABAergic system, Monoaminergic system, Phenolic compounds

## Abstract

*Annona muricata *Linnaeus (Annonaceae), popularly known as *graviola*, is used in folk medicine as both sedative and anticonvulsant. This study correlates the neurochemical profile with the behavioral effects of the hydroalcoholic extract from the leaves of *Annona muricata* (HLEAM) in mice, proposing to elucidate their mechanism of action on the central nervous system. Flavonoids and phenolic compounds were identified and quantified by High Performance Liquid Chromatography (HPLC) method. The acute toxicity (median lethal dose - LD_50_) was determined by probitos method using the percentage of mortality based on the Hippocratic screen. HLEAM (25, 50 and 100 mg/kg) was tested, intraperitoneally (i.p.), in models of sedation, anxiety, motor coordination, and seizures. The endogenous levels of dopamine, norepinephrine and DOPAC were assayed by reverse-phase HPLC with electrochemical detection. The HPLC analysis of the extract revealed the presence of flavonoids (quercetin, isoquercitrin, quercitrin, rutin, and kaempferol) and phenolics acids (gallic, chlorogenic, ellagic and caffeic acids). The LD_50_ was 1091.7 mg/kg and Hippocratic screening indicated central nervous system depressant effect. HLEAM presented sedative effects at doses of 25, 50, and 100 mg/kg, as well as anxiolytic and anticonvulsant effects at a dose of 100 mg/kg. In addition, these effects were partially reversed by flumazenil. The monoamines analysis by HPLC showed that HLEAM decreased the level of norepinefrine and dopamine in the mouse brain striatum. Thus, the results indicate a possible interaction of HLEAM with the GABAergic and monoaminergic systems, adding medicinal value to the popular use of the plant for the treatment of behavioral and neurological disorders.

## Introduction

Central nervous system disorders have become much more frequent in society. Anxiety and depression are currently the most common, being major causes of disability worldwide (1). Thus, the search for new compounds as therapeutic alternatives for the treatment of such disorders has progressed (2). Some plants are well known for their central effects, such as the antidepressant effect of *Hypericum perforatum *L. (Hypericaceae), analgesic effect of *Papaver somniferum *L. (Papaveraceae) (3), antimuscarinic effect of *Atropa belladonna *L. (Solanaceae), and anxiolytic effects of *Piper* *methysticum *G. Forster (Piperaceae), *Passiflora incarnata *L. (Passifloraceae), and *Annona muricata* L. (Annonaceae) (4, 5, 6, 7). Although there are reports of anxiolytic effect for *A. muricata*, there is no research that describes the acute toxicity, the quantification of flavonoids and phenolic acids and the influence of these compounds in the modulation of GABAergics and monoaminergics systems in the central effects of this species.

The Annonaceae family comprises about 135 genera, and over 2,300 species. Native to most tropical or subtropical regions, the genus *Annona *is the most importante and serves as a source of edible fruit. In Brazil, the Annonaceae family presents 26 genera of approximately 260 species (8). Among the species of economic importance, stand out *Annona squamosa *L.*, Annona reticulata* L.*, Annona cherimola *Mill.*, *and *Annona muricata* L., popularly known as graviola (soursop) in Brazil, the fruit is eaten raw, or in the form of juice and ice cream (9). *Annona muricata* L. is described by biologically active compounds, such as alkaloids, flavones, flavonols, flavonones, and tannins (10).

The interest in the species is mainly to the presence of flavonoids and apomorphinic alkaloids that according to some studies have effects on the central nervous system. Ethnopharmacological study reveals the use of dried leaf infusions of *A. muricata* to treat anxiety, insomnia, and headaches (6).

Some studies sought to explain the anxiolytic and sedative activities of *A. muricata*, but there is disagreement among the studies on its possible mechanism of action. Oviedo *et al*. (6) suggests the involvement of the serotonergic system, while Díaz-Véliz and Mora (7) suggests, without further evidence, the involvement of the GABAergic system in both anxiolytic and sedative effects of *A. *muricata. Thus, this research seeks to contribute to the studies already undertaken in order to delineate the possible mechanisms involved in the sedative, anxiolytic, and anticonvulsant effects of hydroalcoholic extract from the leaves of *Annona muricata* L. (HLEAM) in mice.

## Experimental

*Animals*

Female Swiss mices weighing 25 ± 5 g, from the vivarium of the Faculty of Medicine of Estácio de Juazeiro do Norte - FMJ were used. The animals were kept in cages and subjected to a 12 h light/dark cycle, at a constant temperature (24 °C). The animals received food and water *ad libitum*. All experiments were conducted according to the Federal Law No. 11.794/2008; Ethical Principles for Animal Experimentation of the Brazilian College of Animal Experimentation - COBEA and the use of animals was previously approved by the Committee on Animal Use and Experimentation of Regional University of Cariri (URCA) (protocol 23/2012.2).

*Plant material and preparation of hydroalcoholic extract*

The leaves of the *Annona muricata* was collected in August 2011 in Chapada do Araripe (07º 0.143415 south; 039° 24.9330 west), Crato/CE, Brazil. Phd. Maria Arlene Pessoa da Silva (Regional University of Cariri / Department of Biological Sciences / Crato-CE, Brazil) did the vegetal identification. A voucher specimen (#9469) has been deposited at the Herbarium Caririense Dárdano de Andrade-Lima, URCA. The leaves, (180g) were dry and immersed in absolute ethanol and distilled water at a ratio of 1:1 (72 h). The solution obtained was filtered, and the sovent evaporated on a rotary evaporator apparatus. The final product was frozen, and then freeze-dried (yield: 5.8 g). To prepare test solutions, the extract was dissolved in saline solution (NaCl 0.9%). 

*Chemical, apparatus and general procedures*

Pentylenetetrazole was purchased from Sigma® Chemical Co, USA. Ampoules of diazepam, and flumazenil, were purchased from Germed® Brazil and Cristália® Farma Brazil, respectively.

High performance liquid chromatography (HPLC-DAD) was performed with the HPLC system (Shimadzu, Kyoto, Japan), Prominence Auto Sampler (SIL-20A), equipped with Shimadzu LC-20AT reciprocating pumps connected to the degasser DGU 20A5 with integrator CBM 20A, UV-VIS detector DAD (diode) SPD-M20A and Software LC solution 1.22 SP1. All chemical were of analytical grade. Acetonitrile, formic acid, gallic acid, chlorogenic acid, ellagic acid, and caffeic acid purchased from Merck (Darmstadt, Germany). Catechin, epicatechin, quercetin, quercitrin, isoquercitrin, rutin, and kaempferol were acquired from Sigma Chemical Co. (St. Louis, MO, USA). 

*Phytochemical analysis and Quantification of compounds by HPLC*

Phytochemical analysis was performed according to Matos method (11). The tests were based on the visual observation of a change in color or formation of precipitate after the addition of specific reagents, in order to identify the main classes of secondary metabolites in the HLEAM.

To the High Performance Liquid Chromatography (HPLC), reverse phase chromatographic analyses were carried out under gradient conditions using C_18 _column (4.6 mm x 250 mm) packed with 5μm diameter particles. The mobile phase was water containing 1% formic acid (A) and acetonitrile (B), and the composition gradient was: 13% of B until 10 min and changed to obtain 20, 30, 50, 60, 70, 20, and 10% B at 20, 30, 40, 50, 60, 70, and 80 min, respectively (12) with slight modifications. HLEAM was analyzed dissolved in ethanol at a concentration of 20 mg/mL. The presence of eleven antioxidants compounds was investigated, namely, gallic acid, chlorogenic acid, ellagic acid, caffeic acid, catechin, epicatechin, quercetin, quercitrin, isoquercitrin, rutin and kaempferol. Identification of these compounds was performed by comparing their retention time and UV absorption spectrum with those of the commercial standards. The flow rate was 0.7 mL/min, injection volume was 40 μL and the wavelength was 254 nm for gallic acid, 280 nm for catechin and epicatechin, 325 nm for caffeic, ellagic and chlorogenic acids, and 365 nm for quercetin, isoquercitrin, quercitrin, rutin and kaempferol. All the samples and mobile phase were filtered through 0.45 μm membrane filter (Millipore) and then degassed by ultrasonic bath prior to use. Stock solutions of standard references were prepared in the HPLC mobile phase at a concentration range of 0.030–0.250 mg/mL for kaempferol, quercetin, quercitrin, isoquercitrin, catechin, epicatechin and rutin; and 0.030–0.250 mg/mL for gallic, caffeic, ellagic and chlorogenic acids. The chromatography peaks were confirmed by comparing its retention time with those of reference standards and by DAD spectra (200 to 400 nm). Calibration curve for gallic acid: Y = 14286x + 1395.8 (r = 0.9996); catechin: Y = 15097x + 1189.3 (r = 0.9997); epicatechin: Y = 13601x + 1194.5 (r = 0.9992); caffeic acid: Y = 12758x + 1259.7 (r = 0.9996); chlorogenic acid: Y = 13461x + 1275.3 (r = 0.9992); ellagic acid: Y = 13576x + 1346.4 (r = 0.9999); rutin: Y = 12845 + 1305.7 (r = 0.9999); quercetin: Y = 13560x + 1192.6 (r = 0.9991), isoquercitrin: Y = 12873x + 1325.6 (r = 0.9998); quercitrin: Y = 11870x + 1329.8 (r = 0.9993) and kaempferol: Y = 14253x + 1238.9 (r = 0.9997). All chromatography operations were carried out at ambient temperature and in triplicate. The limit of detection (LOD) and limit of quantification (LOQ) were calculated based on the standard deviation of the responses and the slope using three independent analytical curves. LOD and LOQ were calculated as 3.3 and 10 σ/S, respectively, where σ is the standard deviation of the response and S is the slope of the calibration curve (13).

*Acute toxicity and Hippocratic screening*

The general effects of acute administration of HLEAM were evaluated according to a set of signs determined by Almeida *et al.* (14) and Almeida (15). Distinct groups with 4 animals were treated intraperitoneally (i.p.) with HLEAM in the following doses: 10, 50, 100, 500, 1,000, 1,250, 1,500, 1,750, 2,000 mg/kg, and were observed at 10, 30, 60, 120, 180, 240, 360, and 720 min from administration of the drug. The animals were then observed for mortality for 14 days (16). Intraperitoneal route was selected based on the study developed by Díaz-Véliz and Mora (6).

The LD_50_ (median lethal dose) assessment was conducted following the Brazilian rules for acute toxicity of herbal medicines, which seek to identify the median lethal dose. The LD_50_ was calculated by probit analysis using the percentage of mortality (17).

*Behavioral protocols*

All behavioral tests were performed from 12 to 4 p.m., in a quiet and softly illuminated (15 V red light) room, at a constant temperature (23 ± 1 °C) to avoid animal stress. Before the tests, the mice were divided into different groups (n = 9) and treated intraperitoneally (i.p.) with saline or HLEAM and the standard drug of the behavioral model. Thirty min after drug injections, the animals were subjected to the behavioral tests, as described below.

*Open Field Test (OFT)*

The motor activity of the animals was verified on an open field, which consisted of an arena made of clear glass and black tile (30 x 30 x 15 cm), and divided into nine equal quadrants. After 30 min of treatment i.p. with diazepam 1 mg/kg (n = 9), HLEAM (25, 50 and 100 mg/kg) (n = 9/dose), and saline (n = 9), each animal was evaluated for 5 min (18). The parameters measured were: number of crossings (NC), number of groomings (NG-self-cleaning), and the number of rearings (NR-vertical scannings).

*Elevated Plus Maze test (EPM)*

The EPM is based on the model proposed by Pellow *et al.* (19) for rats and validated by Lister (20) for mice. It consists of a device made of wood having two opposing arms open (do not have walls) and are connected to two closed (walled) arms by an intermediary middle square. The maze is elevated 45 cm above the ground.

After 30 min of treatment i.p. with diazepam 1 mg/kg (n = 9), HLEAM (25, 50 and 100 mg/kg) (n = 9/dose), and saline (n = 9), each animal was placed in the center of the apparatus with the head turned towards one of the closed arms. Its behavior was then observed for 5 min, and noted in accordance with the following parameters: number of entries in the open arms (NEOA), time spent in the open arms (TSOA), and number of entries in the closed arm (NECA), and time spent in the closed arms (TSCA). Diazepam (DZP 1 mg/kg, i.p.) was used as a positive control for anxiolytic effect, and flumazenil (2.5 mg/kg, i.p.) was used as benzodiazepine antagonist.

*Rota-rod test (RR)*

Rota-rod test is the most commonly used method for measuring the effect of muscle relaxation or a change in motor coordination possibly caused by drugs (21). For this test, the mice were preselected in a training session 24 h before the experiment. The animals were placed on four legs on a 2.5 cm diameter bar at 25 cm height from the floor. The mice able to remain on the rotating rod at 16 rpm, for 180 s were selected.

In the day test session, the selected animals were treated i.p. with diazepam 2 mg/kg (n = 9), HLEAM (25, 50 and 100 mg/kg) (n = 9/dose), and saline (n = 9), and 30 min afterwards, each animal was tested in the rota rod. The time that an animal spent on the bar (to a one minute maximum) and the number of falls (given three attempts) were recorded (22).

*Pentylenetetrazole-induced seizure test*

After 30 min of treatment i.p. with diazepam 2 mg/kg (n = 9) HLEAM (25, 50 and 100 mg/kg) (n = 9/dose), and saline (n = 9) each animal received pentylenetetrazole (PTZ 80 mg/kg, i.p.). The parameters analyzed were: onset of first seizure (time elapsed between the administration of PTZ until the first clonic or tonic-clonic seizures, in seconds), and the death latency of animals (time of administration of PTZ until the death of the animal, in seconds) (cut-off time: 30 mim.). Flumazenil (5 mg/kg, i.p.) was used as benzodiazepine antagonist.

*Neurochemical protocols*

Endogenous levels of dopamine (DA), norepinephrine (NE), and DOPAC (3,4- dihydroxyphenylacetic acid) were assayed by reverse-phase High Performance Liquid Chromatography (HPLC) with electrochemical detection. Briefly, a C_18_ reverse phase column (Shim-pack, CLC-ODS 150 x 4.6 mm; Shimadzu, Kyoto, Japan), an amperometric detector (Shimadzu, L-ECD-6A), and a liquid chromatography work station were used. Before the test, the mice were divided into two groups containing five animals. After a single intraperitoneal injection of saline (n = 6) and HLEAM 100 mg/kg (n = 6), the mice were sacrificed by cervical dislocation and decapitated.

The brain was removed and quickly dissected to obtain the striatum. Striatum was homogenized in 10% (w/v) of 0.2 M perchloric acid. After centrifugation (14.000 rpm, 30 min), the supernatants were filtered and 20 μL were injected into the (C_18_) column. The mobile phase composed of 15.7 g citric acid, 400 mL of twice-distilled water (pH adjusted to 3.0). The solution was filtered, and 75 mg of sodium octyl sulphate was added. After degasification, 20 mL of acetonitrile, and 10 mL tetrahydrofuran were added to complete a final volume of 500 mL. The flow rate was 0.2 mL/min. The oxidation potential was fixed at 0.85 V using a glass carbon working electrode versus an Ag/AgCl reference electrode. The peak areas of the external standards were used to quantify the sample peaks. The values obtained were expressed as ng/g wet tissue.

*Statistical data analysis*

The results were analyzed by *t* test or ANOVA (followed by the Student-Newman-Keuls test) by GraphPad Prism 5.0 for Windows. Values of *P* < 0.05 were accepted as statistically significant. The LD_50_ was determined through the probitos method (StatPlus® 5.8.) using the percentage of mortality and the logarithm of the dose followed by linear regression.

## Results and Discussion

It is known that the classes and concentrations of plant chemical compounds vary in reason of the biotic and abiotic influences (23), so initially the phytochemical analysis in order to verify if the extract contains bioactive chemical compounds similar described in other extracts. 

Phytochemical analysis by Matos method revealed the presence of various compounds such as alkaloids, flavones, flavonols, xanthones, flavanonols, flavonones, and condensed tannins. Similar results were obtained by Bento* et al.* (10) that identified the presence of flavonoids, alkaloids, and tannins in the leaves of *Annona muricata*, and Oviedo *et al.* (7) detected the presence of flavonoids and alkaloids. Other studies confirmed the presence of flavonoids and alkaloids in other species of the *Annona *genus (9).

**Figure 1:**
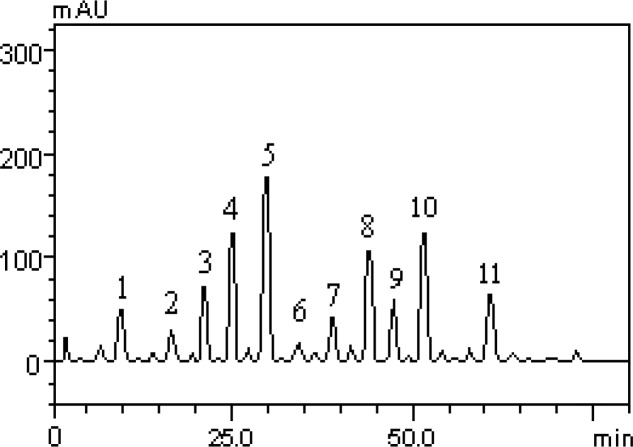
Representative high performance liquid chromatography profile of HLEAM, detection UV was at 325nm. Gallic acid (peak 1), catechin (peak 2), chlorogenic acid (peak 3), caffeic acid (peak 4), ellagic acid (peak 5), epicatechin (peak 6), rutin (peak 7), isoquercitrin (peak 8), quercitrin (peak 9), quercetin (peak 10) and kaempferol (peak 11). Chromatographic conditions are described in the Methods section

However, this is the first study to describe the quantification of flavonoids and phenolic acids of HLEAM by HPLC method. The results revealed the presence of the gallic acid (t_R_ = 10.09 min; peak 1), catechin (t_R_ = 16.32 min; peak 2); chlorogenic acid (t_R_ = 20.57 min; peak 3), caffeic acid (t_R_ = 24.93 min; peak 4), ellagic acid (t_R_ = 29.86 min; peak 5), epicatechin (t_R_ = 34.19 min; peak 6), rutin (t_R_ = 39.05 min; peak 7), isoquercitrin (t_R_ = 44.89 min; peak 8), quercitrin (t_R_ = 47.61 min; peak 9), quercetin (t_R_ = 51.07 min; peak 10) and kaempferol (t_R_ = 61.20 min; peak 11) (Figure 1 and Table 1).

**Table 1:** Quantification of phenolic compounds and flavonoids by high performance liquid chromatography (HPLC) of HLEAM

**COMPOUNDS**	**HLEAM ** **mg/g**	**LOD g/mL**	**LOQ ** **g/mL**
Gallic acid	5.31 ± 0.02 a	0.015	0.049
Catechin	2.79 ± 0.01 b	0.032	0.105
Chlorogenic acid	6.25 ± 0.01 c	0.009	0.029
Caffeic acid	10.14 ± 0.03 d	0.024	0.078
Ellagic acid	13.07 ± 0.02 e	0.013	0.042
Epicatechin	1.83 ± 0.01 f		
Rutin	5.20 ± 0.01 a	0.027	0.090
Isoquercitrin	9.64 ± 0.03 g	0.008	0.026
Quercitrin	6.19 ± 0.01 c	0.035	0.114
Quercetin	10.21 ± 0.02 d	0.019	0.063
Kaempferol	6.27 ± 0.03 c	0.026	0.085

Results are expressed as mean ± standard deviations (SD) of three determinations. Averages followed by different letters differ by Tukey test at *p* < 0.001. Limit of Detection (LOD) Limit of Quantification (LOQ).

Some genera of Annonaceae produce weakly polar flavonoids which facilitate their diffusion of such compounds across the blood-brain barrier and so to act on the central nervous system (24). Some studies indicate that flavonoids quercetin and kaempferol, identified in HLEAM, showed effects sedative (25, 26) which may justify the use of this plant in the relief of anxiety, depression, and sleep disorders. 

In the present study, a pharmacological screening and an acute toxicity test were assessed to evaluate the pharmacological and toxicological profile of HLEAM. The animals treated with HLEAM, i.p., at doses of 500, 1000, 1250, 1500, 1750, and 2000 mg/kg showed central nervous system depressant effect, such as sedation and decreased motility. The behavioral changes were evident 30 min after administration of the extract. At the dose of 1000 mg/kg, 50% of the animals died. With increasing doses of the extract (1250, 1500, 1750 mg/kg) the percentage of mortality increased to 75%. Finally, with the administration of an HLEAM dose of 2000 mg/kg, 100% of the treated animals came to death within 30 min after administration. The LD_50_ was 1091.7 mg/kg, determined by the probit method. In folk medicine, several plants are used in the treatment of psychiatric disorders; this is due to the false belief that by being natural they are safer than synthetic psychotropic medications (6).

However, the use of natural medication does not mean absence of side effects or toxicity; in this aspect, Caparros-Lefebvre *et al*. (27) showed that long-term consumption of *A. muricata* is related to atypical Parkinsonian syndrome. The effect was later associated with annonacin which is an acetogenin presenting toxic activity against cultured dopaminergic mesencephalic neurons (28). The determination of LD_50_ was important for the choice of the dosage range used in this study; thus, it was decided to use dose 2,5%, 5% and 10% below the LD_50_ (25, 50, and 100 mg/kg, respectively).

**Figure 2:**
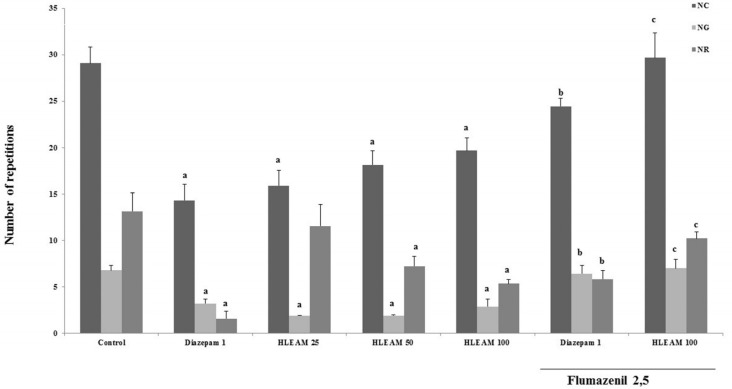
Open Field Test – Effect of HLEAM without and with Flumazenil

As the Hippocratic screening data showed that the HLEAM is endowed with central depressant activity, the animals treated with HLEAM were subjected to specific behavioral tests. In the open field test (Figure 2), HLEAM 25, 50, and 100 mg/kg i.p., significantly reduced NC [(15.89 ± 1.65, 18.11 ± 1.54 and 19.67 ± 1.34, respectively); DF = 6, F (6.65) = 12.99, *P* < 0.0001], NG [(1.88 ± 0.42, 1.88 ± 0.11 and 2.87 ± 0.84, respectively); DF = 6, F(6.57) = 12.20, *P* < 0.0001] and NR [(11.56 ± 2.31, 7.22 ± 1.05 and 5.33 ± 0.47, respectively) DF = 6, F (6.56) = 8.13, *P* < 0.0001], similar to diazepam (14.31 ± 1.71, 3.22 ± 0.49 and 1.55 ± 0.80, respectively) as compared to the respective controls (29.08 ± 1.73, 6.80 ± 0.50 and 13.13 ± 1.99). This suggests that the compound has sedative action, characterized by decreased spontaneous locomotion, possibly by decreasing the excitability of the central nervous system (29).

The bars represent mean standard error of the mean, analyzed by ANOVA, and Student-Newman-Keuls (*post hoc* test); “a”, “b” and “c” vs Control, Diazepam 1 and HLEAM 100, respectively, *P *< 0.05. NC = number of crossings; NG = number of grooming; NR = number of rearing; HLEAM = hydroalcoholic extract from the leaves of *Annona muricata* L.

It is known that both the nigrostriatal and mesolimbic dopaminergic systems play a crucial role in motor control; mesolimbic dopaminergic system, specially, is more closely linked to the control of locomotor activity (30).

Thus, the sedative effect of HLEAM may be due to reduced levels of striatal dopamine (DA) and norepinephrine (NE), as evidenced by the HPLC test (Figure 6). These catecholamines are abundantly present in the central nervous system, and modulate neuronal excitability (31) and drugs such as reserpine, which lead to depletion of catecholamines that may cause sedation, catalepsy, or hypokinesia (32).

The interaction of one or more components of HLEAM with the GABAergic system may be also responsible for the sedative effect observed with the extract. This hypothesis is supported by the fact that the pretreatment with Flumazenil (2.5 mg/kg, i.p. – benzodiazepine antagonist) increased NC, NG, and NR in diazepam (24.45 ± 0.85, 6.44 ± 0.85, and 5.84 ± 0.91, respectively) as well as HLEAM 100 (29.67 ± 2.67 and 7.00 ± 0.96, respectively) treated groups. 

This result can be attributed to the presence of flavonoids identified in the present study (Figure 1), indicating that these substances have neuroactive properties (33) especially, quercetin and kaempferol, which were classified as anxiolytics constituents (25).

Selvakumar (34) had shown that quercetin reduced locomotor activity in the open field test suggesting that in the present study these compounds may be responsible for the anxiolytic and sedative effects.

**Figure 3:**
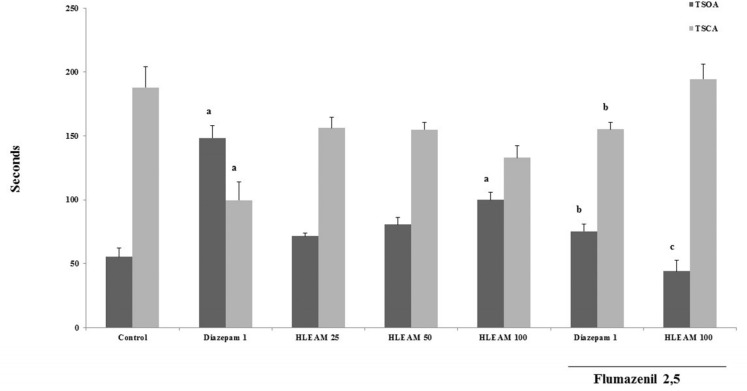
Elevated Plus Maze Test – Effect of HLEAM without and with Flumazenil on the time spent in the open and closed arms

**Figure 4:**
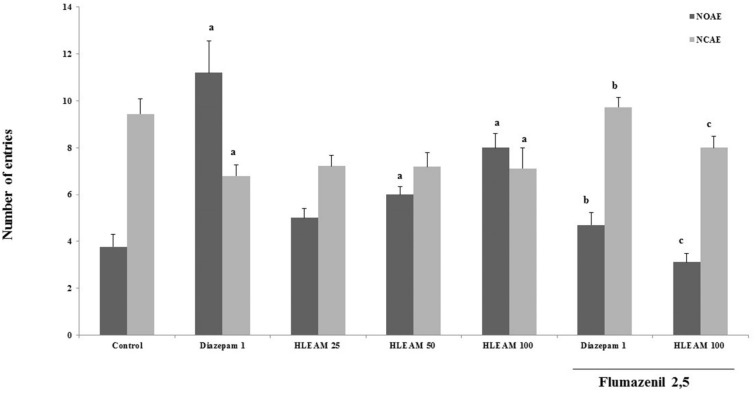
Elevated Plus Maze Test – Effect of HLEAM without and with Flumazenil on the number of entries in the open and closed arms

In the EPM test (Figures 3 and 4), the mice were pre-treated with HLEAM 25, 50, and 100 mg/kg, i.p.; however, only the highest dose of the HLEAM significantly increased NEOA [(5.00 ± 0.40, 6.00 ± 0.33 and 6.00 ± 0.33, respectively) DF = 6, F (6.56) = 16.85, *P* < 0.0001] and TSOA [(71.44 ± 2.66, 80.78 ± 5.52 and 100.0 ± 5.85, respectively) DF= 6, F (6.56) = 24.45, *P* < 0.0001] and decreased TSCA [(156.2 ± 8.31, 155.0 ± 5.80 and 132.9 ± 9.27); DF = 6, F(6.56) = 8.58, *P* < 0.0001], compared to controls (3.77 ± 0.54, 55.25 ± 7.22 and 187.9 ± 16.29, respectively). In the test, diazepam 1 mg/kg, significantly increased NEOA (11.20 ± 1.37) and TSOA (148.5 ± 9.80) and decreased NECA (6.79 ± 0.49) and TSCA (99.67 ± 14.32). Anxiolytic drugs used, especially the benzodiazepines, modulate GABA_A_ receptors (35).

The bars represent mean standard error of the mean, analyzed by )ANOVA( and Student-Newman-Keuls (*post hoc* test); “a”, “b” and “c” vs Control, Diazepam 1, and HLEAM 100, respectively, *P *< 0.05. TSOA = time spent in the open arms; TSCA = time spent in the closed arms; HLEAM = hydroalcoholic extract from the leaves of *Annona muricata* L.

The bars represent mean standard error of the mean, analyzed by ANOVA and Student-Newman-Keuls (*post hoc* test); “a”, “b” and “c” vs Control, Diazepam 1, and HLEAM 100, respectively, *P *< 0.05. NOAE = number of entries in the open arms; NCAE = number of entries in the closed arms; HLEAM = hydroalcoholic extract from the leaves of *Annona muricata* L.

The diazepam presents anxiolytic effect by increasing the number of entries (and time spent) in the open arms, reducing at the same time, the number of closed arm entries and time spent. Administration of HLEAM developed a pattern of effects similar to that observed with the diazepam. Elevated plus maze test is considered as an important experimental model for the study of drugs with anxiolytic effects of the benzodiazepine type (36). 

This observation may indicate that the anxiolytic-like effect of HLEAM was due to the interaction of one or more of the extract constituents with the GABA_A_ receptors. Wasowski (37) showed that some types of flavones possess selective affinity for the benzodiazepine binding site on the GABA_A_ receptors in the brain. This finding confirms the study of Díaz-Vélez and Mora (6) which interconnects the anxiolytic effects of *Annona muricata* (0,5g/v.o.) to the GABAergic system.

To investigate the possibility that a lower dose tested by Diaz-Velez and Mora (6), possibly modulates GABA_A_ receptors, the HLEAM 100 mg/kg was associated with 2.5 mg/kg flumazenil, a benzodiazepine antagonist with affinity for the GABA_A_ receptor (38). Pretreatment with flumazenil partially reversed the effect of both, diazepam, and HLEAM in the elevated plus maze, by decreasing the NEOA (4.67 ± 0.52 and 3.11± 0.38, respectively) and TSOA (75.32 ± 5.86 and 44.14 ± 8.31, respectively) and increasing the NECA (9.73 ± 0.42 and 8.00 ± 0.5, respectively), and TSCA (155.1 ± 5.79 and 194.4 ± 12.15, respectively), significantly in relation to the group treated with HLEAM or diazepam alone.

Benzodiazepines have several effects related to modulation of GABA_A_ receptor, such as sedation, reduced muscle tone, and diminished motor coordination; this latter is a common feature of many neurological disorders and pharmacological intoxication (39, 40). The benzodiazepines compromise the execution of activities that require reflexes and motor control by inhibiting polysynaptic reflexes and internuncial transmission, and in high doses can depress transmission at the skeletal-neuromuscular junction (41). The rota-rod test is used to evaluate the effect of drugs on motor coordination, allowing detection of neurological disorders including ataxia, sedation, and muscle relaxation, in addition to neurotoxicity (42, 43). 

In the rota-rod test, HLEAM (at all doses) had no effect on motor coordination of the animals (data not shown). This observation is important because despite its sedative and anxiolytic effect, the HLEAM, at the doses tested, did not promotes muscle either relaxation or loss of reflexes, the unwanted effects commonly seen with benzodiazepine drugs.

Once the HLEAM presented central depressant effect, the anticonvulsant effect by pentylenetetrazole-induced seizures model was investigated. The mechanisms of seizure, activation, propagation, and maintenance are poorly understood. Seizures can be characterized as clinical manifestations resulting from abnormal neuronal discharges, producing over-excitation of the neurons, and also occur by imbalances between the mechanisms of inhibitory and excitatory neurotransmission (44).

**Figure 5:**
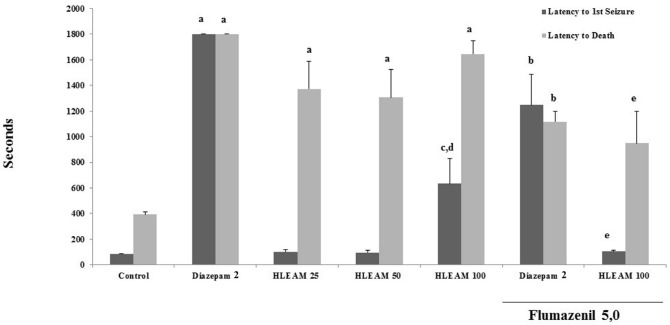
Pentylenetetrazole-induced seizures test – Effect of HLEAM without and with Flumazenil

The mice were pre-treated with HLEAM 25, 50, and 100 mg/kg i.p.; however, only the highest dose of HLEAM showed significant increased latency until the first seizure [(102.3 ± 17.59, 97.44 ± 14.03, and 633.9 ± 194.0, respectively); DF = 6, F (6.56) = 59.91, *P* < 0.0001], when compared to the control (87.00 ± 1.46). On the other hand, HLEAM 25, 50 and 100 mg/kg increased significantly the death latency parameter [(1374 ± 215.6, 1307 ± 215.6 and 1646 ± 101.6, respectively); DF = 6, F (6.56) = 8.39, *P* < 0.0001], as well as diazepam 2 mg/kg, i.p. (1800 ± 0.0) compared to the control (393.7 ± 19.91) (Figure 5).

The bars represent mean standard error of the average, analyzed by (ANOVA) and Student-Newman-Keuls (*post hoc* test); “a”, “b”, “c”, “d” and “e” vs Control, Diazepam 2, HLEAM 25, HLEAM 50 and HLEAM 100, respectively, *P *< 0.05. HLEAM = hydroalcoholic extract from the leaves of *Annona muricata* L.

According to Oviedo *et al. *(7) *A. muricata* did not confer protection against electroshock or pentylenetetrazol-induced seizures. The difference may be related to dosages used or to absorption differences due to the route of administration (in the Oviedo *et al.* study, the extract was administered orally). The effect observed in this reasearch may be due to the action of the extract on the GABAergic system. When associated with flumazenil 5 mg/kg, HLEAM 100 mg/kg decreased the first convulsion and death latency of the animals (105.8 ± 9.07 and 949.8 ± 248.6, respectively) compared to the group treated with HLEAM 100 mg/kg only. Similarly, the association flumazenil 5 mg/kg and diazepam 1 mg/kg decreased the first convulsion and death latency of the animals (1248 ± 238.3 and 1117 ± 80.91, respectively), when compared to the group treated with diazepam 1 mg/kg alone.

**Figure 6:**
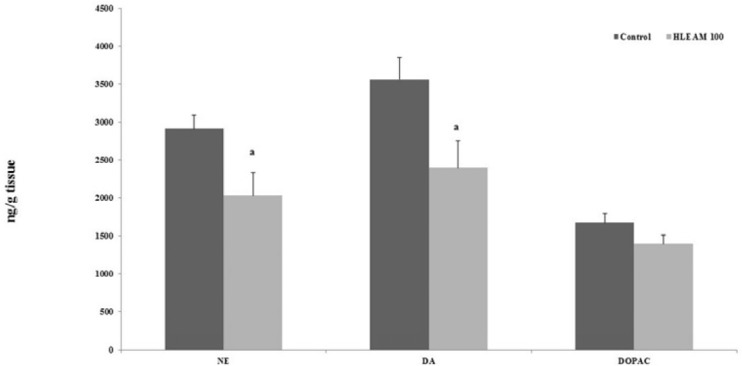
High Performance Liquid Chromatography (HPLC) – Effect of HLEAM treatment on striatic monoamine and metabolite contents

In Figure 6 the administration of HLEAM caused a significant decrease in the levels of NE [2032 ± 297.3; DF = 4, DF (3.3) = 2.74, P = 0.044] and DA [2396 ± 350.8; DF = 4, F (3.3) = 1.48, *P* = 0.042], compared to their respective controls (2912 ± 179.3 and 3559 ± 287.7). The concentration of DOPAC [1397 ± 115.5; DF = 4, F (3.3) = 1.21, *P* = 0.152] did not change with HLEAM administration, compared to the control group (1673 ± 122.3). In the present study, HLEAM showed a neuroprotective effect, although less than the diazepam in PTZ-induced seizure test. Shouse *et al*. (45) asserted that the increase in noradrenaline and serotonin, (but not dopamine), would be accompanied by electroencephalogram changes, due to intermittent neuronal discharges in the amygdala, (and/or the locus coeruleus), and/or widespread throughout the brain cortex during the seizure.

The bars represent mean standard error of mean, analyzed by test T (unpaired test); “a” vs Control, *P *< 0.05. NE = norepinephrine; DA = dopamine; DOPAC = 3,4-dihydroxyphenylacetic acid; HLEAM = hydroalcoholic extract from the leaves of *Annona muricata* L..

Thus, the neuroprotective effect of HLEAM may be also due to the reduction in noradrenaline concentration as was evidenced by the high performance liquid chromatography study.

## Conclusion

The HLEAM showed sedative, anxiolytic and anticonvulsant-like effects similar to benzodiazepine drugs, associated with decreased neuronal excitability by modulating the activity of the GABA_A_ receptor. This hypothesis is proven when HLEAM actions are antagonized by flumazenil. In addition, the results indicate a possible interaction of extract with the monoaminergic system.

The central effects reported justifying the traditional use of the plant for treating behavioral and neurological disorders.
